# Altered Dairy Protein Intake Does Not Alter Circulatory Branched Chain Amino Acids in Healthy Adults: A Randomized Controlled Trial

**DOI:** 10.3390/nu10101510

**Published:** 2018-10-15

**Authors:** Utpal K. Prodhan, Amber M. Milan, Eric B. Thorstensen, Matthew P. G. Barnett, Ralph A. H. Stewart, Jocelyn R. Benatar, David Cameron-Smith

**Affiliations:** 1 Liggins Institute, The University of Auckland, 85 Park Road, Grafton, Private Bag 92019, Auckland 1023, New Zealand; u.prodhan@auckland.ac.nz (U.K.P.); a.milan@auckland.ac.nz (A.M.M.); e.thorstensen@auckland.ac.nz (E.B.T.); 2 Department of Food Technology and Nutritional Science, Mawlana Bhashani Science and Technology University, Tangail 1902, Bangladesh; 3 Riddet Institute, Palmerston North 4442, New Zealand; matthew.barnett@agresearch.co.nz; 4 Food Nutrition & Health Team, AgResearch Limited, Private Bag 11008, Palmerston North 4442, New Zealand; 5 The High-Value Nutrition National Science Challenge, Auckland-1023, New Zealand; 6 School of Medicine, Faculty of Medical and Health Sciences, The University of Auckland, 85 Park Road, Grafton, Private Bag 92019, Auckland 1023, New Zealand; ralph.stewart@auckland.ac.nz (R.A.H.S.); jbenatar@adhb.govt.nz (J.R.B.); 7 Green Lane Cardiovascular Service, Auckland City Hospital, Auckland 1030, New Zealand; 8 Food & Bio-Based Products Group, AgResearch Limited, Private Bag 11008, Palmerston North 4442, New Zealand

**Keywords:** amino acids, protein, insulin sensitivity, randomized controlled trial

## Abstract

Dairy, as a major component of a high protein diet, is a critical dietary source of branched chain amino acids (BCAA), which are biomarkers of health and diseases. While BCAA are known to be key stimulators of protein synthesis, elevated circulatory BCAA is an independent risk factor for type 2 diabetes mellitus. This study examined the impact of altered dairy intake on plasma BCAA and their potential relationship to insulin sensitivity. Healthy adults (*n* = 102) were randomized to receive dietary advice to reduce, maintain, or increase habitual dairy intake for 1 month. Food intake was recorded with food frequency questionnaires. Self-reported protein intake from dairy was reported to be reduced (−14.6 ± 3.0 g/day), maintained (−4.0 ± 2.0 g/day) or increased (+13.8 ± 4.1 g/day) according to group allocation. No significant alterations in circulating free amino acids (AA), including BCAA, were measured. Insulin sensitivity, as assessed by homeostatic model assessment-insulin resistance (HOMA-IR), was also unaltered. A significant change in dairy protein intake showed no significant effect on fasting circulatory BCAA and insulin sensitivity in healthy populations.

## 1. Introduction

The recommended dietary allowance (RDA) of protein for the general adult population has been established to be 0.8 g protein per kilogram of body weight [1]. At this level of intake, all essential amino acids (EAA) and sufficient amine groups required for adequacy of non-essential amino acids (NEAA) are generally met by a mixture of dietary protein sources in adults at energy balance [1]. However, there is increasing evidence that consumption of protein above the RDA can promote weight regulation, maintenance of muscle mass, with reduced risk for type 2 diabetes mellitus (T2DM) and cardiovascular disease (CVD) [2]. In contrast, other studies demonstrate the capacity for adverse actions of modest to high protein diets (greater than 15% of total energy) on liver function [3], and a graded total cancer risk with increasing protein [4]. Conversely, high protein diets (greater than 24% of total energy intake) during calorie restricted weight loss may be beneficial in attenuating bone loss [5].

To more comprehensively understand the health implications of differing levels of protein intake, studies must address the dynamics and biological functions of proteins at the level of amino acids (AA). This is because increased circulatory free AA, predominantly the branched chain amino acids (BCAA), are present in metabolic disease states, and associated with insulin resistance and T2DM [6,7,8,9]. Additionally, disturbances in circulatory AA are strong metabolic predictors of heightened disease risk [10], as is evident in longitudinal studies where higher circulating concentrations of BCAA are predictive of a 1.3 to 3-fold greater risk of metabolic disease, T2DM [11,12] and stroke [13].

Few studies have investigated whether dietary protein intake is important in the regulation of circulatory AA levels, including those of BCAA. Although most dietary AA are degraded in the liver, BCAA are unique in that catabolism occurs primarily in muscle [14]. Hence, dietary BCAA intake may impact the concentrations of BCAA in plasma [15,16]. Current dietary guidelines for many countries make specific reference for the inclusion of dairy foods as a component of a healthy diet [17,18]. In the western population, the contribution of dairy to protein intake is approximately 16% of total intake [19]. While dairy foods are a rich source of high-quality protein, dairy is particularly rich in BCAA (approximately 20% of total protein) [20,21]. Alterations in dairy intake may therefore result in changes in the circulatory concentrations of BCAA. However, there is limited data from randomized controlled trials measuring the impact of modifying dairy intake on plasma circulating BCAA levels in healthy adults. Thus, the aim of the current study was to determine whether altered dairy intake impacts circulatory AA, including BCAA. Furthermore, we investigated whether this dietary change impacted on insulin sensitivity concurrent with changes in circulatory BCAA concentrations.

## 2. Materials and Methods

### 2.1. Subject Selection

This study reports plasma amino acid and insulin sensitivity, from a previously published randomized parallel intervention; the primary outcomes reported the impact of the dietary intervention on cardio-metabolic risk factors [22] and plasma fatty acid profiles [23]. In brief, healthy volunteers (*n* = 180) living in Auckland, New Zealand regularly consuming dairy and who were willing to modify dairy intake for one month were recruited by advertisement from February 2011 to September 2011. Exclusion criteria included an inability to tolerate dairy food, known diabetes, cardiovascular disease, inflammatory conditions, currently taking any lipid or glucose modifying medication and age ≤18 years. Ethics approval was obtained from the Northern X Ethics Committee and all participants provided written informed consent. This study was registered with the Australian New Zealand Clinical Trials Registry (ID: ACTRN12612000574842).

**Figure 1:**
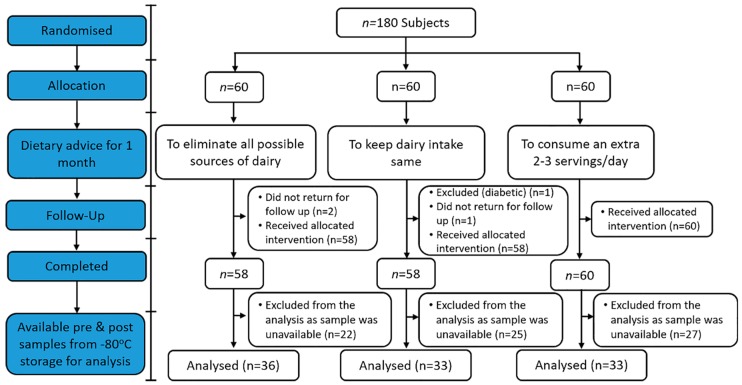
Flow diagram of the study participants from eligibility criteria screening to study completion.

After the primary analyses were complete [22,23], a secondary analysis was undertaken on samples from 102 individuals (21–80 years) for which remaining pre- and post-intervention plasma samples were identified in storage. Participant screening, enrolment and randomization, including the number of samples available for this analysis, is presented in Figure 1.

### 2.2. Study Design and Treatments

Following enrolment, subjects were assigned equally across intervention arms using a computer-generated random allocation sequence.

Weight, height, and waist circumference were measured according to the International Standards for Anthropometric Assessment [24] while dietary intake of all dairy sources was recorded during the preceding 3 days at baseline and over the last 3 days of the one month intervention, using the National Cancer Institute Diet History Questionnaire [25], a validated food frequency questionnaire (FFQ). Questions on dairy and meat intake were unchanged but those related to alcohol, fruits, vegetables, grain and sweeteners were not included. This shortened FFQ was not separately validated. The individual frequencies and serving sizes of the dairy foods were converted into absolute intake of high fat milk (mL), low fat milk (mL), or high fat dairy solids (g). From these intakes, mean daily protein intake (g/day) for each subject, was calculated using the Food and Nutrition Database of Food Standards Australia New Zealand [26]. Dairy serving sizes were defined using the United States Department of Agriculture (USDA) criteria [27].

Participants were provided with detailed dietary instruction on how to alter dietary intake to either increase or decrease dairy intake. Researchers and participants were not blinded to group allocation as this was discussed with participants; however, investigators who assessed the outcomes were blinded until all analyses were completed. Participants randomized to decrease dairy were asked to eliminate all possible sources of dairy; however, alternatives such as rice milk or soya were suggested. Participants randomized to maintain dairy were asked to keep their dairy intake same, whereas the increased dairy intake group was counselled to consume an extra two to three servings of dairy per day. Advice was given to consider all forms of dairy, including full- and skimmed varieties of liquid milk, fermented dairy products, ice-cream and cheeses. Cheese products from cow, sheep and buffalo were included. Chocolate and snack products containing dairy (including cheese snacks) were not included in this advice. The research coordinator conducted a telephone interview at 2 weeks to aid in maintaining compliance. As previously reported, dietary compliance [28] has been reported on the basis of corresponding changes in the plasma concentrations of ruminant-derived pentadecanoic acid (C15:0) and heptadecanoic acid (C17:0) [23].

### 2.3. Biochemical Analysis

Fasting blood samples were taken from all participants at baseline (pre-intervention) and after 1 month (post-intervention). Blood was collected in plain, heparin, and EDTA tubes. Heparinised blood tubes were centrifuged (2000× *g*, 10 min, 4 °C) and the separated plasma was analysed for glucose within 60 min. Blood in the plain tubes was allowed to clot, the tubes were then centrifuged (2000× *g*, 10 min, 4 °C) and the resulting serum stored at −80 °C for later insulin analysis. EDTA tubes were held at 4 °C and centrifuged (2000× *g*, 10 min, 4 °C) within 20 min of the blood draw; the resulting plasma samples were continuously stored at −80 °C, with no free-thaw cycles prior to free AA analysis.

Fasting plasma glucose and lipids were analysed using a standard Roche Modular analyser (Roche, Mannheim, Germany) with the following methods-glucose: glucose oxidase; total cholesterol (TC): cholesterol oxidase; High-density lipoprotein cholesterol (HDL-c): PEG-modified cholesterol esterase and oxidase, with dextran sulphate; triglyceride (TG): lipoprotein lipase and oxidase. Low-density lipoprotein cholesterol (LDL-c) was calculated using the Friedwald equation. Serum insulin was measured by chemiluminescence immunoassay using an Abbott Architect analyser (Abbott Laboratories, Abbott Park, IL, USA). Insulin sensitivity was measured as HOMA-IR and was calculated from fasting glucose and insulin levels using a standard formula [29]. Plasma free AA were assessed by ultra-high pressure liquid chromatography (UPLC) following a standard protocol [30] with some modification. Briefly, 20 µL of plasma were mixed with sodium tungstate and acid extracted with L-nor-valine as an internal standard. After centrifuging at 4 °C, 10 µL of the supernatant was added to 70 µL borate buffer (0.2 M pH 8.8) and reacted with 10 µL 6-aminoquinolyl-*N*-hydroxysuccinimidyl carbamate reagent. The mixture was then heated for 10 min at 55 °C before being injected onto a UPLC system and fluorescence detection (Thermo Scientific Dionex Ultimate 3000 pump; Thermo Fisher Scientific, Dornierstrasse, Germany) with a Kinetex EVO C18 1.7 µm 150 × 2.1 mm column. Data capture was for each 36 min chromatographic run using Chromeleon 7.1 software (Thermo Fisher Scientific). Plasma AA concentrations were calculated from standard curves generated for each AA using mixed standards (Sigma Chemical Company, St. Louis, MO, USA). Three plasma samples were included in each batch for quality control. The mean (range) overall coefficient of variation for the amino acids was 6.7% (2.7–13.9%). For some participants, it was not possible to assess the concentration of arginine (*n* = 3), or lysine (*n* = 4) accurately because of poor signal saturation of the fluorescence detector in the UPLC; thus, no data were available. No samples had concentrations below the lowest or above the highest standard limit of quantification.

### 2.4. Statistical Analysis

For the current study, all available samples (33–36 subjects per group) from previously reported analysis [22] were analysed. Baseline characteristics and dietary intake patterns were compared across the three groups using one-factor ANOVA. Two-factor (time and dietary intervention) repeated-measures ANOVA followed by Sidak post hoc tests were used for all multiple comparisons between groups using SPSS Statistics 24 (IBM Corp., Armonk, New York, NY, USA). α was set at 0.05. Statistical outliers were identified and removed using three times the interquartile range (IQR). The heatmap was created with R software (version R i386 3.2.2) using the packages gplots (ggplot2) (R Development Core Team, R Foundation for Statistical Computing, Vienna, Austria). Unless otherwise stated, data are represented as means ± SEMs.

## 3. Results

There were no significant differences in baseline characteristics by dietary group (Table 1). The mean age of the population was 46 ± 1 years and 70% of the participants were female. The average body mass index (BMI) was 24.8 ± 0.4.

At baseline, no differences in dairy intake (servings/day) and dairy protein intake (g/day) in any of the intervention groups (*p* > 0.05) were observed as assessed by FFQ; however, a significant change was observed in dairy intake (*p* < 0.001) and dairy protein intake (*p* < 0.001) during the course of the intervention (Table 2). When compared between the groups, both dairy intake and dairy protein intake were higher in the increased dairy intake group than the reduced dairy intake group (*p* < 0.05 each, respectively) only. Compared to their respective baseline intake, significant differences in dairy intake and dairy protein intake were achieved in the increased dairy intake group and reduced dairy intake group (*p* < 0.01). However, for dairy intake, the amount change (servings/day) achieved in the increased dairy intake group was found to be significantly different from the other two groups (*p* ≤ 0.01) though the difference between reduced dairy intake group and maintained dairy intake group was not significant (*p* > 0.05). Likewise, for dairy protein intake, the amount change (g/day) achieved in the increased dairy intake group was found to be significantly different from other two groups (*p* ≤ 0.01) and also the difference between reduced dairy intake group and maintained dairy intake group was significant (*p* < 0.05). Participants were not insulin resistant with HOMA-IR of 1.41 ± 0.10 with no baseline differences between the groups (*p* > 0.05), and also after the month-long intervention, there were no significant differences in HOMA-IR between the groups (*p* > 0.05; Table 3).

**Figure 2:**
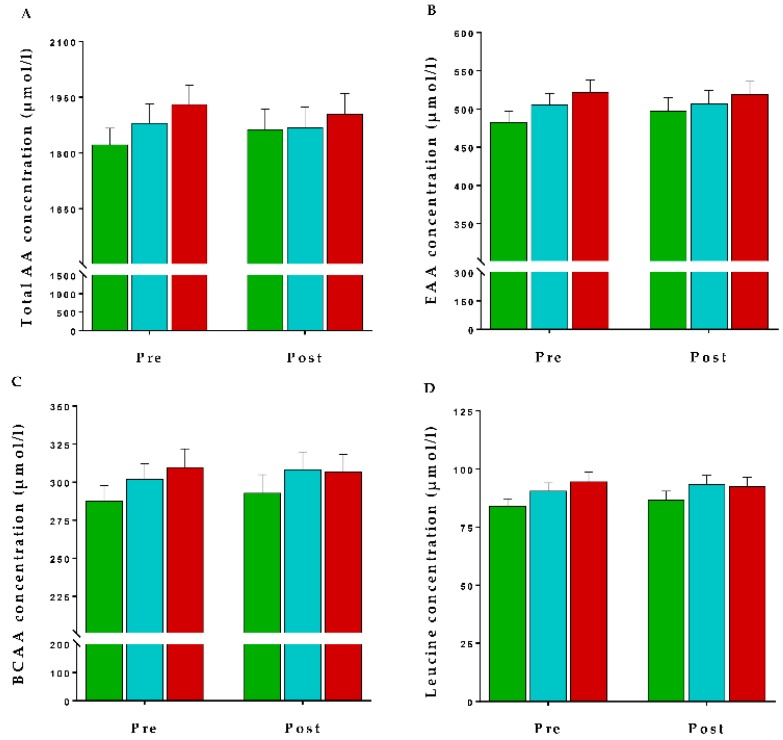
Plasma free amino acid concentrations of the three dairy intake groups (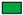: Reduced, 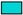: Maintained, and 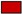: Increased) across the intervention period. Values are presented as mean ± SEM of (**A**) total amino acids (TAA), (**B**) essential amino acids (EAA), (**C**) branched chain amino acids (BCAA) and (**D**) leucine concentrations (µmol/L). Comparisons between dairy intake groups and interactions (time x dietary intervention group) analyzed by two-factor repeated-measures ANOVA. None of the changes were significant (*p* > 0.05).

Similarly, there were no alterations in plasma total amino acids (TAA), BCAA, EAA or leucine concentrations, at the completion of the intervention (*p* > 0.05 each, respectively; Figure 2).

**Figure 3:**
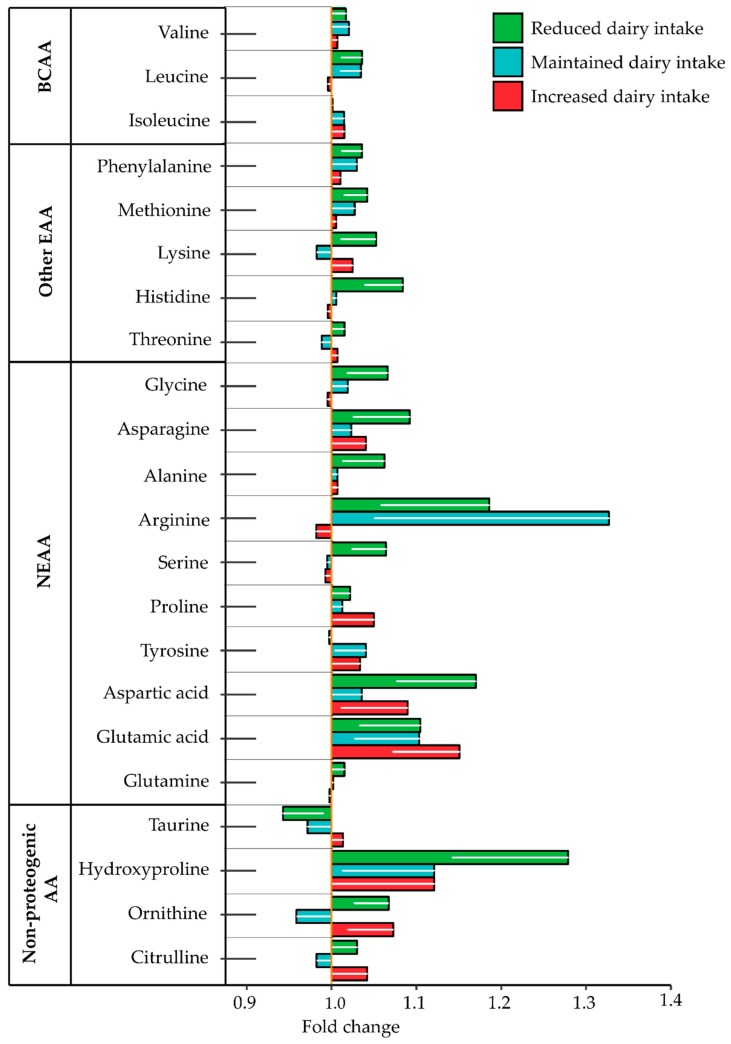
The influence of altered dairy intake on amino acid concentrations as fold change observed in all the three dairy intake groups compared to their baseline for the branched chain amino acids (BCAA), essential amino acids (EAA), non-essential amino acids (NEAA) and non-proteogenic amino acids (AA). Values are presented as mean ± SEM. Comparisons between dairy intake groups and interactions (time × dietary intervention group) analyzed by two-factor repeated-measures ANOVA. None of the changes was significant (*p* > 0.05 each, respectively).

No individual free AA concentrations were altered by the intervention (*p* > 0.05 each, respectively); these non-significant changes are shown in Figure 3, details of the changes in amino acid concentrations of the three dairy intake groups are presented in the Table S1.

## 4. Discussion

Dairy food is a major dietary source of protein and BCAAs [31]. Recent studies have demonstrated an adverse relationship between increased circulatory BCAAs and measures of insulin sensitivity [6,32,33]. However, little data exist on whether increased dairy protein (and BCAA) intake may impact circulatory plasma BCAA concentrations and whether this may in turn influence insulin sensitivity in healthy adults. Consistent with the dairy intake of the New Zealand adult population [18], the cohort in this study had a baseline average dairy food intake of 2.6 servings per day. A difference of 3.9 servings per day was reported between those advised to restrict dairy intake and those who were given dietary advice to increase intake which resulted in an additional intake of 28.4 g dairy protein in the ‘increased dairy intake’ group relative to the ‘reduced dairy intake’ group. These results suggest the absence of a major effect of dairy protein intake on circulatory BCAA levels in free-living healthy adults in response to altered dairy intake for one month.

The ingestion of dairy foods has been shown to result in a postprandial rise in plasma free AAs, including the BCAAs, and the kinetics of this response is dependent upon the constituent proteins within the dairy foods [34,35]. Despite the transient nature of postprandial AA dynamics, notable changes in circulating AAs have been described following short-term dietary increases in BCAA-rich foods in young children [36]. However, no effect of dietary BCAA changes on circulating concentrations has likewise been reported [37], consistent with the current study. Furthermore, while a causative effect of BCAAs on metabolic alterations has been proposed [7,38], more recent research has instead suggested that the altered BCAA profiles associated with metabolic dysfunction are rather symptomatic of the alterations in BCAA catabolism observed in insulin resistant states [39]. Indeed, catabolic intermediates of BCAA metabolism were recently found to correlate with improvements in insulin sensitivity, rather than changes in circulating BCAA [40]. Therefore, consistent with other studies [37,40,41], the modifications in dairy protein intake achieved in the current study may have had no impact on the homeostatic regulation of circulating AA during post-absorptive periods.

While this study addressed the impacts of alterations in dairy foods, unique differences between differing protein-rich foods exist in relation to satiety [42], rate of digestion [43] and impact on microbial function [44]. Thus, it is also possible that other major protein sources, such as red meat or plant-based proteins may exert differential actions on circulating BCAA and insulin sensitivity. Arguing against this is the observation that circulatory AAs, including BCAAs, has been shown to not be altered in a differing patterns of protein intake, including meat-eaters, fish-eaters, vegetarians and vegans [45].

This study was conducted using healthy subjects, predominantly female, with a self-directed change in dietary intake. While dairy intake was reported to be different between groups, self-reported dietary recall has known shortcomings [46]. Previous analysis from this study has reported correlations between plasma odd-chain fatty acids and self-reported dairy intake, supporting intervention compliance [23]. While this study did not quantify the intake of all other possible sources of dietary BCAAs, it was demonstrated that other major protein sources, such as red meat, were not altered [22]. The study evaluated healthy volunteers in part to reduce confounding effects of disease and treatments which influence metabolic risk, yet female predominance may limit the generalizability of the findings to the general population.

## 5. Conclusions

A substantial change in habitual BCAA intake from dairy foods, approximately equivalent to the amount of BCAAs found in 1.5 to 2 servings of milk per day, for 1 month did not affect circulating BCAA plasma concentrations or insulin sensitivity in healthy individuals. This suggests that among healthy adults, variation in dairy intake is not a major determinant of circulating BCAA levels. Further studies examining the impact of dietary protein intake, including dairy ingestion, on circulatory AAs and metabolic health in individuals with pre-existing insulin sensitivity, with evidence of altered fasting plasma amino acids, may be warranted.

## Figures and Tables

**Table 1:** Baseline subject characteristics.

	Reduced Dairy Intake	Maintained Dairy Intake	Increased Dairy Intake
*N*	36	33	33
Female (%)	75	72	64
Age (Years)	47 ± 2	46 ± 2	47 ± 2
Systolic Blood Pressure (mmHg)	116 ± 2	115 ± 2	114 ± 3
Diastolic Blood Pressure (mmHg)	70 ± 2	70 ± 2	72 ± 2
BMI	24.4 ± 0.9	23.3 ± 0.9	25.5 ± 0.9
TC (mmol/L)	5.43 ± 0.17	5.24 ± 0.17	5.18 ± 0.17
HDL (mmol/L)	1.79 ± 0.08	1.71 ± 0.09	1.69 ± 0.09
LDL (mmol/L)	3.08 ± 0.15	3.04 ± 0.16	2.98 ± 0.16
TG (mmol/L)	1.09 ± 0.13	1.11 ± 0.13	1.12 ± 0.13
Glucose (mmol/L)	5.34 ± 0.08	5.29 ± 0.08	5.32 ± 0.09
Insulin (mU/L)	5.7 ± 0.8	5.9 ± 0.7	6.6 ± 0.8

Values represent mean ± SEM. BMI: body mass index; TC: total cholesterol; HDL: high-density lipoprotein; LDL: low-density lipoprotein; TG: triglyceride; TC, HDL, LDL, TG and glucose were measured from plasma whereas insulin was measured from serum; *p* > 0.05 for all.

**Table 2:** Changes in dairy intake and dairy protein intake for each randomized group throughout the intervention period.

	Reduced Dairy Intake	Maintained Dairy Intake	Increased Dairy Intake	*p*-Value
**Dairy intake (servings/day)**				
Baseline	3.0 ± 0.4	2.6 ± 0.3	2.1 ± 0.2	0.218 ^a^
Post intervention	1.2 ± 0.4 ^†^	2.1 ± 0.2	4.4 ± 0.8 *^†^	<0.001 ^b^
Change	−1.6 ± 0.4	−0.5 ± 0.2	+2.3 ± 0.8 ^#^	<0.001 ^c^
**Dairy protein intake (g/day)**				
Baseline	24.6 ± 3.3	22.0 ± 2.3	18.0 ± 1.9	0.208 ^a^
Post intervention	9.9 ± 3.1 ^†^	18.0 ± 1.9	31.8 ± 3.9 *^†^	<0.001 ^b^
Change	−14.6 ± 3.0 ^ф^	−4.0 ± 2.0	+13.8 ± 4.1 ^#^	<0.001 ^c^

Values represent mean ± SEM. ^a^ Changes between dairy intake groups at baseline analyzed by one-factor ANOVA; ^b^ comparisons between dairy intake groups and interactions (time × dietary intervention group) analyzed by two-factor repeated-measures ANOVA; ^c^ comparisons between three dairy intake groups performed with baseline adjusted one-factor ANOVA. * Indicates a significant difference (*p* < 0.05) relative to the reduced dairy intake group; ^#^ indicates a significant difference (*p* ≤ 0.01) relative to both the reduced dairy intake and maintained dairy intake groups; ^ф^ indicates a significant difference (*p* < 0.05) between the reduced dairy intake and maintained dairy intake groups; ^†^ indicates significant difference (*p* < 0.01) relative to baseline.

**Table 3:** Changes in HOMA-IR for each randomized group after the intervention period.

	Reduced Dairy Intake	Maintained Dairy Intake	Increased Dairy Intake	*p*-Value
**Insulin Resistance (HOMA-IR)**				
Baseline	1.36 ± 0.14	1.41 ± 0.18	1.57 ± 0.21	0.734 ^a^
Post intervention	1.38 ± 0.14	1.62 ± 0.16	1.58 ± 0.18	0.390 ^b^
Change	−0.14 ± 0.10	+0.17 ± 0.14	−0.02 ± 0.22	0.398 ^c^

Values represent mean ± SEM. ^a^ Changes between dairy intake groups at baseline analyzed by one-factor ANOVA; ^b^ comparisons between dairy intake groups and interactions (time × dietary intervention group) analyzed by two-factor repeated-measures ANOVA; ^c^ comparisons between three dairy intake groups performed with baseline adjusted one-factor ANOVA.

## Supplementary Materials

The following is available at http://www.mdpi.com/2072-6643/10/10/1510/s1, Table S1: Plasma concentrations of amino acids by dairy intake groups throughout the intervention period.
