# Supplementary material for: Altered Dairy Protein Intake Does Not Alter Circulatory Branched Chain Amino Acids in Healthy Adults: A Randomized Controlled Trial
Source: Nutrients. 2018 Oct 15;10(10):1510. doi: 10.3390/nu10101510 (PMC6213722; doi:10.3390/nu10101510)
Supplement: Supplementary file 1 [file nutrients-10-01510-s001.pdf]

Table S1. Plasma concentrations of amino acids by dairy intake groups throughout the intervention period.

	Reduced dairy intake (n=36)		*Maintained dairy intake (n=33)*		*Increased dairy intake (n=33)*		*p-value*[a]
	**Baseline**	**Post intervention**	**Baseline**	**Post intervention**	**Baseline**	**Post intervention**	
Branched-chain amino acids							
**Valine**	163.45 ± 5.39	166.14 ± 5.99	168.9 ± 5.63	171.61 ± 6.26	172.84 ± 5.63	172.32 ± 6.25	0.82
**Leucine**	83.97 ± 3.49	86.74 ± 3.72	90.62 ± 3.64	93.42 ± 3.89	94.63 ± 3.64	92.61 ± 3.89	0.32
**Isoleucine**	40.16 ± 1.91	40.05 ± 1.99	42.69 ± 2.00	43.38 ± 2.08	42.32 ± 2.00	41.99 ± 2.08	0.84
Other essential amino acids							
**Phenylalanine**	36.41 ± 1.05	37.48 ± 1.04	38.49 ± 1.10	39.33 ± 1.08	38.81 ± 1.10	38.23 ± 1.08	0.52
**Methionine**	19.01 ± 1.13	19.79 ± 1.29	19.97 ± 1.18	20.53 ± 1.34	20.1 ± 1.18	19.99 ± 1.35	0.64
**Lysine**	97.86 ± 3.85	101.18 ± 4.29	101.84 ± 4.10	98.1± 4.56	103.75 ± 3.96	104.81 ± 4.42	0.44
**Histidine**	23.75 ± 0.85	25.37 ± 1.24	25.26 ± 0.89	25.35 ± 1.30	26.29 ± 0.89	26.05 ± 1.30	0.44
**Threonine**	21.15 ± 0.92	21.2 ± 0.92	21.45 ± 0.96	20.91 ± 1.00	23.21 ± 0.96	23.11 ± 0.97	0.80
Non-essential amino acids							
**Glycine**	186.79 ± 12.57	195.69 ± 12.41	192.48 ± 13.13	188.43±12.96	203.89 ± 13.13	187.10 ± 12.96	0.36
**Asparagine**	35.84 ± 2.44	37.65 ± 2.66	39.11 ± 2.55	40.22 ± 2.77	38.0 ± 2.55	38.51 ± 2.77	0.90
**Alanine**	244.53 ± 9.71	252.41 ± 10.95	258.54 ± 10.15	256.37 ±11.44	257.46 ± 10.15	253.69 ± 11.44	0.69
**Arginine**	16.96 ± 1.72	17.5 ± 1.91	18.73 ± 1.85	19.33 ± 2.06	20.3 ± 1.82	18.22 ± 2.03	0.33
**Serine**	78.51 ± 4.10	82.49 ± 4.09	72.58 ± 4.29	70.57 ± 4.27	75.14 ± 4.29	73.77 ± 4.27	0.16
**Proline**	143.03 ± 7.46	142.54 ± 7.87	152.89 ± 7.79	154.73 ± 8.22	165.51 ± 7.79	168.43 ± 8.22	0.92
**Tyrosine**	43.44 ± 1.97	42.52 ± 2.01	47.46 ± 2.06	48.29 ± 2.10	45.63 ± 2.06	46.41 ± 2.10	0.68
**Aspartic acid**	1.93 ± 0.19	1.86 ± 0.15	1.96 ± 0.20	1.79 ± 0.15	1.89 ± 0.20	1.98 ± 0.15	0.61
**Glutamic acid**	21.41 ± 1.81	22.63 ± 1.97	22.47 ± 1.89	23.39 ± 2.06	22.2 ± 1.89	24.78 ± 2.06	0.72
**Glutamine**	393.7 ± 9.93	399.3 ± 10.39	394.86 ± 10.34	393.9 ± 10.85	411.7 ± 10.37	406.54 ± 10.85	0.61
Non-proteogenic amino acids							
**Taurine**	54.01 ± 2.60	48.84 ± 2.39	46.74 ± 2.71	43.37 ± 2.44	46.49 ± 2.71	46.04 ± 2.44	0.41
**Hydroxyproline**	6.83 ± 0.71	7.39 ± 0.67	6.78 ± 0.74	6.37 ± 0.70	6.91 ± 0.74	6.57 ± 0.70	0.66
**Ornithine**	32.56 ± 1.87	34.11 ± 1.78	34.80 ± 1.96	31.19 ± 1.86	32.94 ± 1.96	34.38 ± 1.86	0.06
**Citrulline**	80.22 ± 4.08	81.44 ± 4.09	86.98 ± 4.26	84.76 ± 4.28	81.73 ± 4.26	80.76 ± 4.28	0.82

Values represent mean ± SEM. [a] Comparisons between dairy intake groups and interactions (time x dietary intervention group) analyzed by two-factor repeated-measures ANOVA. None of the changes were significant (p>0.05 each, respectively).
